# Current status of research on composite bipolar plates for proton exchange membrane fuel cells (PEMFCs): nanofillers and structure optimization

**DOI:** 10.1039/d3ra08054d

**Published:** 2024-02-28

**Authors:** Li Wenkai, Xie Zhiyong, Zeng Haodong

**Affiliations:** a Central South University Changsha 410017 Hunan Province China xzy507@csu.edu.cn

## Abstract

The proton exchange membrane fuel cell (PEMFC) is an efficient and clean energy source with promising applications. However, drawbacks such as high cost and low durability limit its application. Bipolar plates are an important component of PEMFCs, which not only account for a large proportion of the price and quality, but also affect the performance and durability of PEMFCs. Unlike traditional graphite and metal bipolar plates, composite bipolar plates have the advantages of easy processing, low cost, and corrosion resistance, but their lower performance limits their practical applications. This paper firstly summarizes the current research progress of various nanofillers used to improve the performance of composite bipolar plates, and discusses the improvement of the performance of composite bipolar plates by different forms and types of nanofillers. The morphological characteristics of different types of nanofillers and their effects on the improvement of conductive pathways are also analyzed. Subsequently, the means of structural optimization of composite bipolar plates are summarized, and specific optimization methods for phase interface, graphite/resin dispersion morphology, and conductive network structure are discussed in detail. Finally, challenges are discussed. Overall, this review can provide a reference for future research on composite bipolar plates.

## Abstract 2

Research progress and analysis of composite bipolar plates for proton exchange membrane fuel cells – nanofillers: carbon black, carbon nanotubes, graphene. Structural optimization: distribution, conductive path and interface modification.

## Graphical abstract

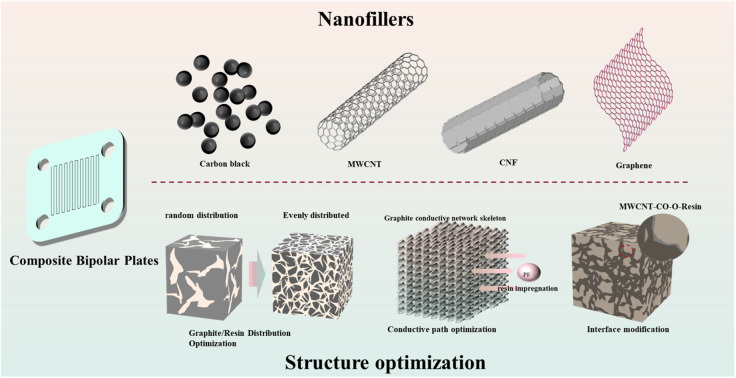

## 1 Introduction

Since non-renewable fossil energy reserves are limited and environmentally unfriendly, tackling this problem requires the development of new types of energy for the sake of long-term development and the global environment.^1–4^ Many kinds of new energy sources have been developed, such as solar energy,^5^ wind energy^6^, bio-energy,^7^ geothermal energy,^8^ and so on. Fuel cells, as one of the alternatives to non-renewable fuels, convert the chemical energy in the reaction process into electric power,^9^ which has the advantages of a high energy conversion rate,^1,10,11^ less pollution,^12^ no mechanical vibration, low noise^13^ and so on. A proton exchange membrane fuel cell is a kind of fuel cell, and its power generation principle is: under the action of catalysts, hydrogen and oxygen separated by a proton exchange membrane react at the two poles of the cell to produce water and at the same time produce electricity.^14–16^ Because of the characteristics of the reaction itself, the proton exchange membrane fuel cell has a high energy conversion rate, is easy to use, and can be used in various applications.^17^ As a result of all of the above, PEMFC has come a long way in the past and is still attracting a high level of interest.^18–20^ However, due to shortcomings such as high cost and low durability,^21–23^ its practicalization is still slow. Therefore, reducing the cost and increasing the usefulness of PEMFC components is a problem that needs to be solved today.^24–26^

**Fig. 1:**
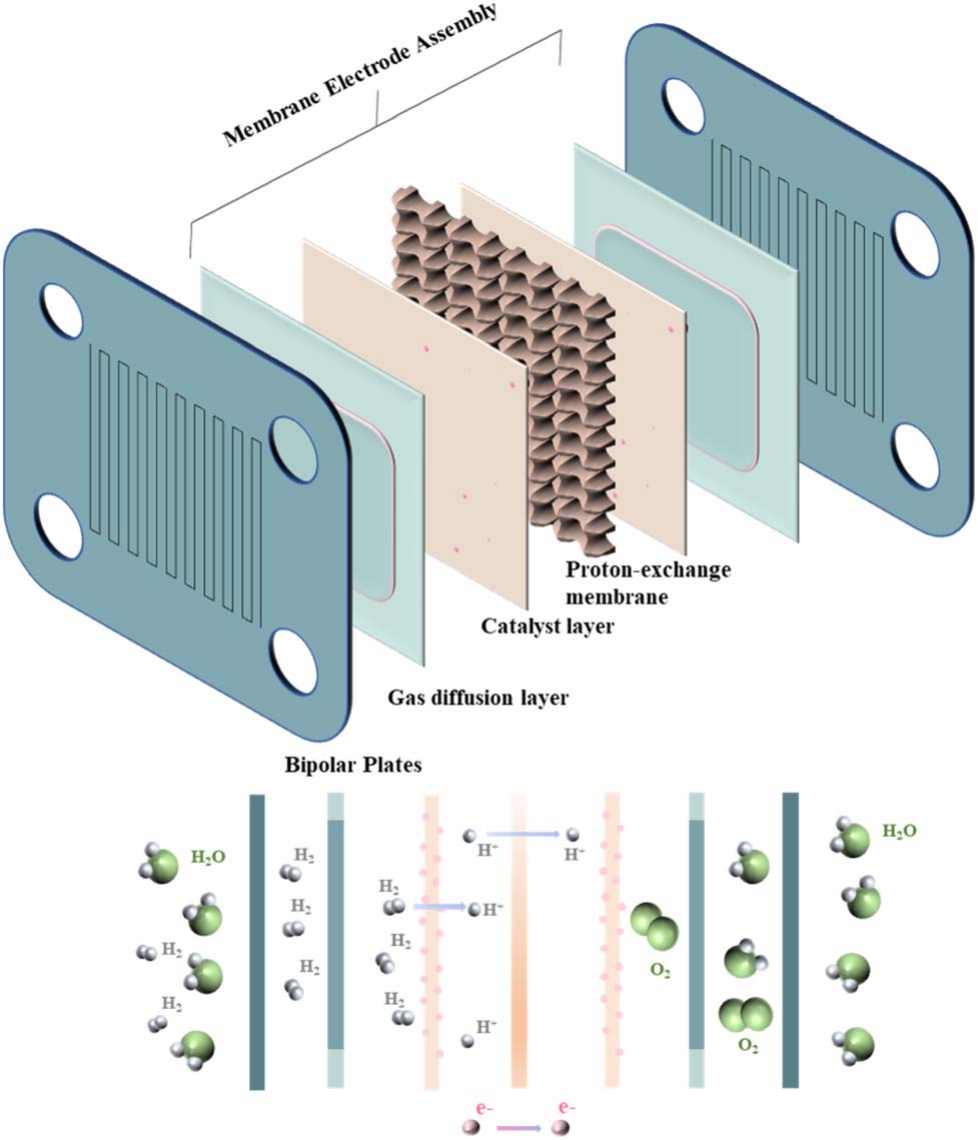
Composition and reaction principle of PEMFC.

The PEMFC consists of membrane electrode assemblies (MEA), gas diffusion layers (GDL), and bipolar plates (BPs).^27,28^ As shown in Fig. 1, BPs, as one of the essential constituent structures of PEMFC, have a crucial influence on the performance of PEMFC^29^ since BPs assume the following roles in the operation of PEMFC: (1) managing the flow of water and heat generated during PEMFC reactions.^30^ (2) Transportation of the raw materials needed for the reaction: hydrogen and oxygen^31^ (3) supporting membrane electrodes and connecting single-cell conduction currents.^32^ In addition, the quality and cost of BPs, as the main structure of the PEMFC, also account for a sizable portion of the PEMFC.^33–35^ In addition, the bipolar plate should also act as a gas barrier to avoid gas interactions in the reaction. This is because gas crossover and leakage will not only lead to the mixing of reactants, which is detrimental to the safety of the cell, but will also lead to the inability of the bipolar plate to deliver the gas stably and uniformly to the GDL, which reduces the efficiency of the reaction. Therefore, to improve PEMFC performance and reduce costs, BPs need better mechanical properties, better electrical conductivity, better thermal conductivity, lower gas permeability, better corrosion resistance, and lower cost and lighter weight.^36,37^Table 1 shows the performance requirements set by the U.S. Department of Energy to advance the utility of BPs.^38,39^

**Table 1:** (Ref. 38 and 39) Department of Energy (DOE) requirement for bipolar plates in 2025

Property	Unit	Requirement
Electrical conductivity	S cm^−1^	>100
Areal specific resistance	Ω cm^2^	<0.01
Hydrogen permeability	cm^3^ s^−1^ cm^−2^	<2 × 10^−6^
Contact resistance	Ω cm^2^	<0.01
Corrosion current density	μA cm^−2^	<1
Cost	$·per kW	<5
Flexural strength	MPa	>45

The research of BPs has been developed over the years and can be mainly categorized into three types according to their preparation materials: graphite bipolar plates, metal bipolar plates, and graphite–resin composite bipolar plates.^40–45^ Bipolar plates of different materials have different advantages and disadvantages: graphite bipolar plates have better electrical and thermal conductivity and meager contact resistance with the GDL,^46^ but the mechanical properties of graphite BPs are poor, and the cost of processing them into complex shapes is high.^47,48^ As the earliest developed class of bipolar plates, graphite bipolar plates have a mature preparation process.^49^ Metal is one of the ideal materials for bipolar plates due to its excellent machinability, mechanical properties and electrical conductivity. However, metal bipolar plates are prone to corrosion and the metal ions generated during the corrosion process may damage other components of the PEMFC, and due to the poor corrosion resistance consequently caused by lower durability.^50,51^ In addition, pure metal bipolar plates face the problem of hydrogen embrittlement, *i.e.*, the degradation of mechanical properties due to long-term absorption of hydrogen. Currently, corrosion resistance of metal bipolar plates is improved and hydrogen absorption is mitigated by anticorrosive coatings and other methods, which also increase the contact resistance and manufacturing costs.^52,53^ Also, ions produced during the corrosion of the metal may damage the proton exchange membrane. Composite bipolar plate is a polymer/carbon composite bipolar plate using resin as the matrix and carbon materials as the conductive additive. Among them, the reinforcing phase mainly adopts high strength polymer materials, such as phenolic resin (PF), polyethersulfone (PES), polypropylene (PP), polyimide (PI), polyetheretherketone (PEEK), polyphenylene sulfide (PPS), *etc.*, while carbon materials are most widely used in graphite materials, such as expanded graphite, natural graphite, synthetic graphite, spherical graphite, *etc.*, and carbon fibers, hard carbon, and other materials have also been used. As a kind of new type of bipolar plate with increased research heat in recent years, it combines the advantages of metal bipolar plate and graphite bipolar plate: easy to be processed,^54^ good corrosion resistance, and low cost,^36,55,56^ but due to the problems of poor compatibility between the resin and the graphite, the electrical conductivity and the mechanical properties are difficult to be balanced, and the porosity is large. In order to solve the problem of poor performance of composite bipolar plates,^57,58^ the current primary use by adding conductive fillers, such as carbon nanotubes (CNTs),^59,60^ graphene,^60^ carbon fibers,^61–63^ carbon black,^64–67^*etc.* Among these conductive fillers, nanofillers have become the main second conductive filler due to their excellent performance and significant effect, often used together with graphite to enhance the performance of composite bipolar plates. However, the excessively high specific surface energy makes nanofillers have disadvantages such as easy agglomeration, so their incorporation needs to be carefully considered, or else they will do the opposite. Selection of suitable types of nanofillers and reasonable addition process are the current considerations. Another means to enhance the performance of composite bipolar plates without compromising the electrical conductivity and mechanical properties is structural optimization. Through the overall optimization of the graphite–resin bulk and interfacial structures^65,68^ or adding nanofillers,^55,59,60,64–67,69^ the electrical conductivity under the premise of ensuring mechanical properties can be increased so that the composite bipolar plates have balanced electrical conductivity and mechanical properties. In summary, all three types of bipolar plates with different substrates have their own disadvantages and advantages. Consequently at this stage of research, in order to improve the overall performance of the PEMFC, it is very important to improve the performance of the bipolar plate by various means to make up for its shortcomings.^70–72^

**Fig. 2:**
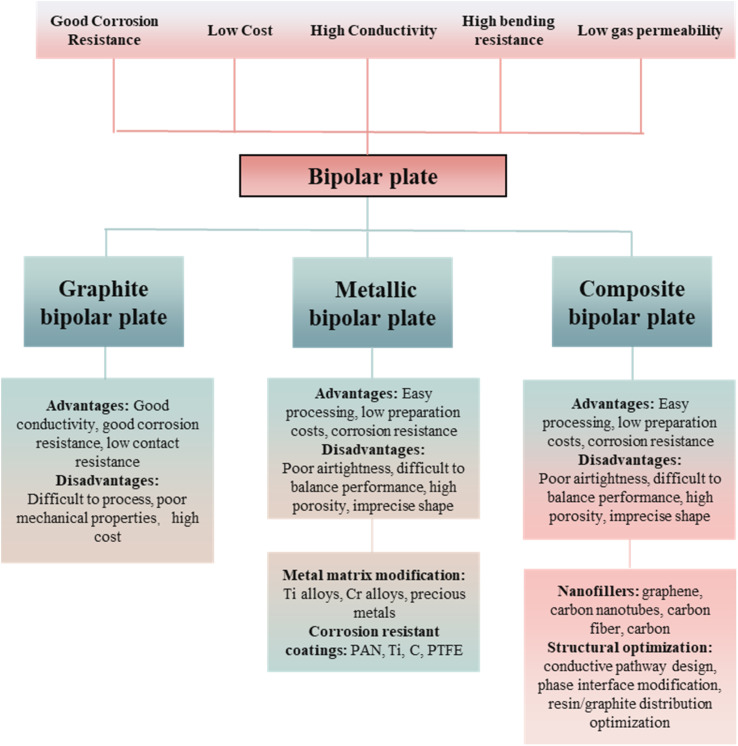
Bipolar plate performance needs and research directions at a glance.^36,37,47–51,55–67^

In this review, the current research on composite bipolar plates is summarized and analyzed mainly from the perspectives of nanofillers and structural optimization of composite bipolar plates as Fig. 2 shows. At the same time, based on the current research status, this review also provides researchers with information on the current research trends in composite bipolar plates. It compares the advantages and disadvantages of different methods for improving the performance of composite bipolar plates.

## 2 Application of nanofillers in composite bipolar plates

Nanofillers, especially carbon-based nanofillers, have a wide range of applications in the field of bipolar plates, which can be used not only for the modification of metal bipolar plate coatings,^73^ but also for the enhancement of the performance of composite bipolar plates.

For composite bipolar plates, if the performance is regulated by adjusting the ratio of raw materials, *i.e.*, changing the ratio between graphite and resin raw materials, a problem is encountered. As the graphite content rises, the electrical conductivity of the composite bipolar plate rises, but the mechanical properties will decline. While most of the conductive nanofillers can enhance the conductivity more substantially without compromising the mechanical properties too much, some can also enhance the mechanical properties through filling and other effects.^74,75^ Therefore, adding nanofillers is a feasible approach at this stage to solve the problem of insufficient comprehensive performance of composite BPs.

In the research of composite bipolar plates in recent years, a variety of nanofillers and micron fillers have been shown to positively affect the performance of composite BPs. Among them, carbon-based fillers are the more widely studied ones, such as: (1) multi-walled carbon nanotubes (MWCNTs),^59,60,76^ (2) graphene,^60,76–78^ (3) carbon nanofibers (CNFs),^55,59^ (4) conductive carbon black (CBs).^64–67,79,80^ It is worth mentioning that, unlike nanoscale conductive fillers, micron-sized carbon fibers are often used in composite bipolar plates not as additives but as the main body of the conductive phase, which plays a role similar to that of graphite.^47,61,63,81–85^ Most nanofillers have excellent intrinsic conductivity, which can not only fill the gaps between the particles of composite BPs, but also improve the conductive pathway in the composite BPs, so they can enhance the electrical conductivity of the composite BPs without decreasing the mechanical properties. Some nanofillers can also enhance the mechanical properties of the composite BPs through the pore filling and reinforcement effects.

However, nanofillers also have their limitations: nanofillers are challenging to be uniformly dispersed in the matrix of the composite bipolar plates due to their small particle size, which gives them a high surface energy. In addition, because most of the carbon-based nanofillers have poor compatibility with resins,^86–88^ excessive addition of nanofillers can not be wetted by resin. It may cause problems such as increased porosity and destruction of the internal structure of the bipolar plates. Therefore, how to use nanofillers rationally and give full play to their performance is what needs to be done at this stage.

This subsection will categorize and elaborate on several well-used and hotly researched mainstream nanofillers, including their characteristics, advantages, and disadvantages.

### 2.1 Carbon black

Carbon black (CB) is a 0-dimensional, granular carbon-based nanofiller. Due to its small size and point-like form, carbon black is commonly used as a diffusion reinforcing phase in composites to improve the mechanical properties of the composites. As a carbon-based material, CB inherently has superior electrical conductivity and is therefore well suited for the preparation of composite bipolar plates where both electrical conductivity and mechanical properties are required.

CB has been considered one of the reinforcing fillers for PEMFCs since long ago. Kim M. *et al.*^89^ studied composite bipolar plates for carbon fiber/PF and found that the addition of carbon black effectively improved the body resistivity and surface contact resistance of the composite bipolar plates, and at an addition of 4%, the carbon black reduced the body resistance by 36% and the interfacial contact resistance by 27%. Antunes R. A. *et al.*^90^ prepared CB/synthetic graphite (SG)/PVDF composite bipolar plates using carbon black as a conductive filler and investigated the effect of CB addition on the structure and corrosion resistance. The results showed that adding carbon black can effectively improve the performance. However, when the carbon black exceeds 5%, it will lead to a decrease in performance due to the increase of porosity, which may be caused by the poor compatibility of carbon black with resin, and a large amount of carbon black will lead to the decrease of compaction of composite bipolar plates because of the poor wettability.

**Fig. 3:**
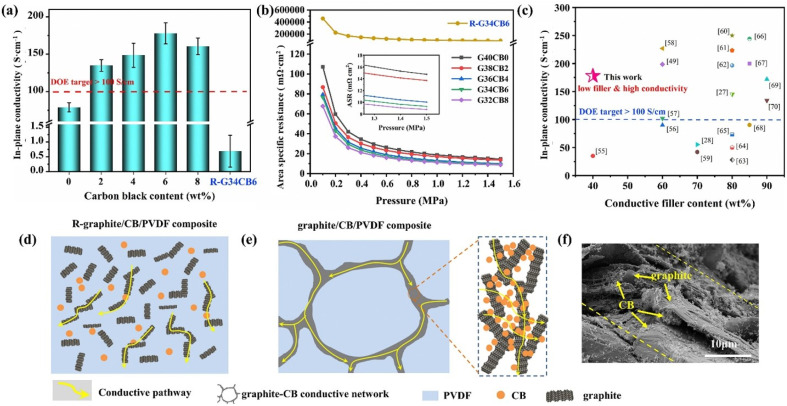
(a) In-plane conductivity and (b) area resistivity of composite bipolar plates, (c) comparison of the electrical properties of G/CB/PVDF composite BP with those of other literature, (d) conductive pathway with randomly distributed fillers, (e) synergistic conductive pathway of graphite/CB, (f) morphology of composite bipolar plates.^64^

Hu B. *et al.*^64^ prepared a CB/PVDF/EG composite bipolar plate with a synergistically polarized conductive network structure, where CB is selectively distributed in the graphite phase, which together constructs the overall conductive network structure. The specific preparation method is to mix graphite with carbon black to form a graphite–carbon black hybrid powder and then mix it with resin. Since graphite and carbon black directly have a more solid bond, unlike the traditional melt blending method that leads to random distribution of fillers, the carbon black obtained by this method tends to be distributed in the graphite phase, as shown in Fig. 3. Thanks to its designed structure, the performance of carbon black is fully utilized, and the composite bipolar plate has a significant performance improvement, with a conductivity of 177.87 S cm^−1^, a flexural strength of 49.16 MPa, and a power density of 646.08 mW cm^−2^. As shown in Fig. 3, the 0-dimensional granular CB fills the interstices of the conductive pathways composed of graphite, resulting in a complete and continuous conductive network structure inside the composite.

**Fig. 4:**
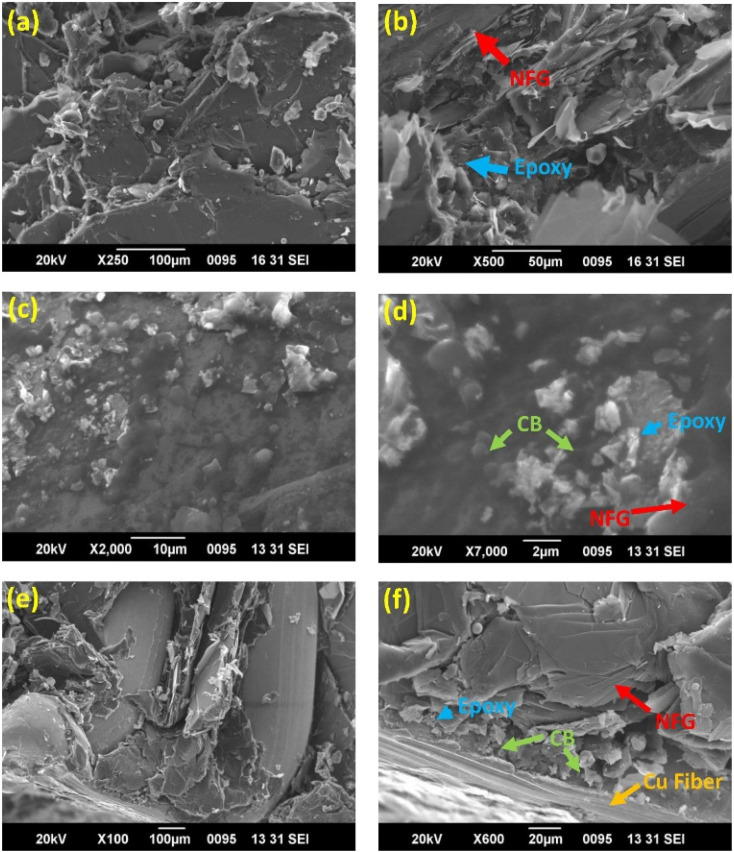
SEM images of the cross-sectional areas of composite bipolar plates at different scales: (a) SEM image of E40/G60 cross-section (b) E40/G60 enlarged cross-section (c) E40/G50/CB10 cross-section (d) E40/G50/CB10 enlarged cross-section (e) cross-section of E40/G42/CB10/Cu8 (f) enlarged cross-section of E40/G42/CB10/Cu8.^65^

Mathew C.^65^*et al.* prepared a copper-fiber-filled composite bipolar plate by using EP and natural flake graphite (NFG) as a conductive filler and adding carbon black as an auxiliary filler. As shown in Fig. 4, CB particles existed in the middle of the voids of NFG. The specific surface area of the conductive filler was greatly expanded due to the addition of CB. The distance between the conductive fillers became small enough to conduct electricity through the tunneling mechanism. In addition, the addition of copper fibers could also connect the dispersed conductive pathways in series to enhance the conductivity of the composite bipolar plate. As a result, the bipolar plates exhibited good electrical conductivity (169 S cm^−1^), as well as a large bending strength (43 MPa).

**Fig. 5:**
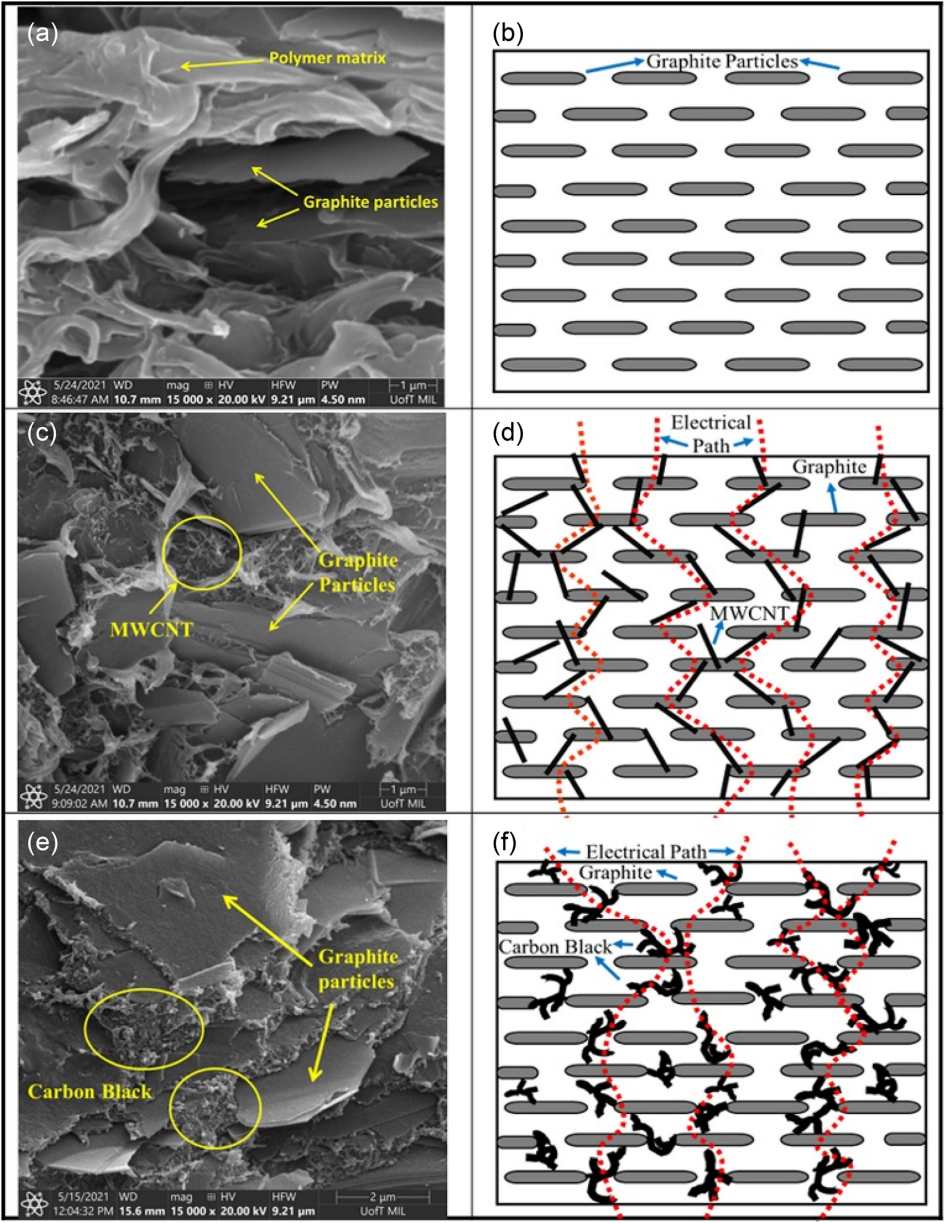
SEM micrographs and schematic diagrams of PP composites; graphite (a and b), MWCNTs/graphite (c and d) and (e and f) carbon black/graphite.^66^

Tariq M. *et al.*^66^ compared the different effects of MWCNTs and CB as conductive fillers on the properties. They investigated the mechanism of their enhancement using G/PP composite bipolar plates as an example. MWCNTs tend to enhance the conductivity by connecting the conductive fillers. In contrast, CB enhances the conductivity by filling the gaps between the particles, as shown in Fig. 5. Moreover, the overall trend of the two fillers on the properties is roughly similar, with the conductivity of the composite bipolar plates rising with the rise of nanofillers. In contrast, the mechanical properties decrease due to wetting and agglomeration. By reasonably optimizing the filler ratio, the conductivity of composite bipolar plates can reach 49.25 S cm^−1^ with the addition of 2% MWCNTs and 83% graphite, but the mechanical properties are relatively poor. In comparison, a relatively balanced feedstock ration of both properties was observed in the case of 82.5 wt% graphite and 2.5 wt% CB with a conductivity and flexural strength of 25.7 S cm^−1^ and 34.8 MPa, respectively.

Krause B. *et al.*^67^ investigated the effect of CB as a conductive filler instead of graphite on the performance of composite bipolar plates. The CB/G/PP composite bipolar plates were prepared by melt mixing, which allowed the filler to be fully dispersed and then molded. The addition of CB significantly reduced the resistivity of the composite bipolar plates, and its volume resistivity could be reduced to 0.12 Ω cm^2^.

In general, the research on the application of CB in composite bipolar plates is still high at this stage, and most of the results show that CB can effectively improve the various properties of composite bipolar plates. However, the trend of performance change after CB addition is similar to that of MWCNTs, and excessive addition will lead to cost increase and performance decrease, so rational dispersion of CB by some means or improvement of the surface properties of CB to solve the problems of agglomeration of CB and difficulty of being wetted by the resin is noteworthy in the following research stage.

### 2.2 MWCNTs

Unlike conductive carbon black, carbon nanotubes are a one-dimensional nanoscale filler with a large aspect ratio curled from single or multi-layer graphene, so it has excellent mechanical properties, electrical conductivity, and thermal conductivity. It is often used as a performance enhancer for composite materials. Multi-walled carbon nanotubes (MWCNTs) are carbon nanotubes with slightly lower properties and larger diameters than single-walled carbon nanotubes. However, because the preparation cost of multi-walled carbon nanotubes is lower than that of single-walled carbon nanotubes, they are often considered as additives for composites such as bipolar plates, which require more cost considerations.

**Fig. 6:**
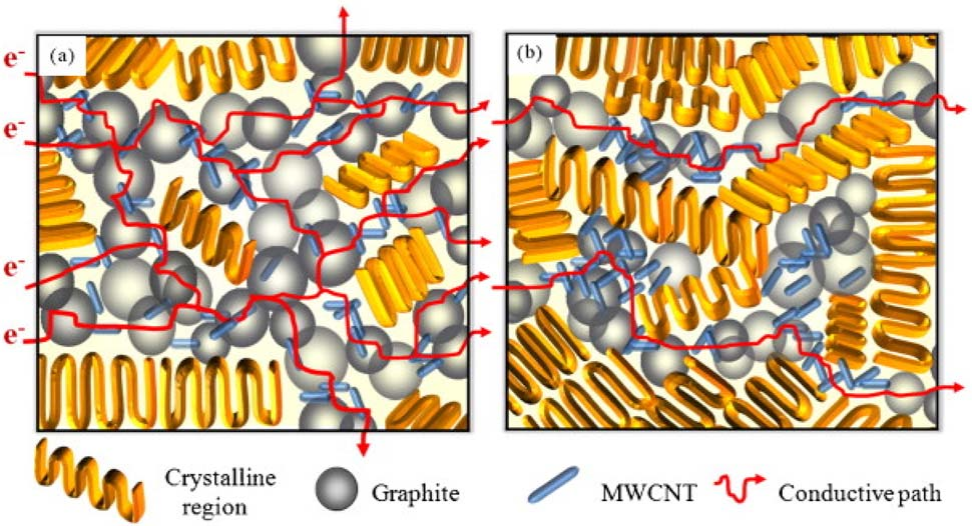
MWCNTs synergistically construct a conductive network inside the composite bipolar plate.^91^

Multi-walled carbon nanotubes (MWCNTs), as a new type of nanofillers, have been considered as one of the conductive fillers for PEMFCs in the past, and sufficient studies have been carried out. Liao S. H. *et al.*^91^ prepared MWCNT/PP/graphite(G) composite bipolar plates and investigated the dispersion of carbon nanotubes and the role of carbon nanotubes within the composite material after their incorporation. As shown in Fig. 6. The results showed that MWCNTs demonstrated potential in improving the performance of the composite bipolar plates, and the in-plane conductivity of the prepared composite bipolar plates reached up to 548 S cm^−1^ due to the synergistic effect of MWCNTs, and at this point, the flexural strength was also enhanced to more than 30 MPa. Akhtar M. N. *et al.*^92^ prepared a composite bipolar plate using natural graphite (NG), epoxy resin (EP), and MWCNTs. Thanks to the phenomenon of possible synergistic electron transfer effect between MWCNTs and NGs, the composite bipolar plate has excellent electrical conductivity and is able to be multi-loaded with EP while the electrical conductivity is satisfied, and therefore, the mechanical properties are improved. The composite bipolar plates loaded with MWCNTs exhibit DOE-compliant conductivity up to 129 S cm^−1^.

**Fig. 7:**
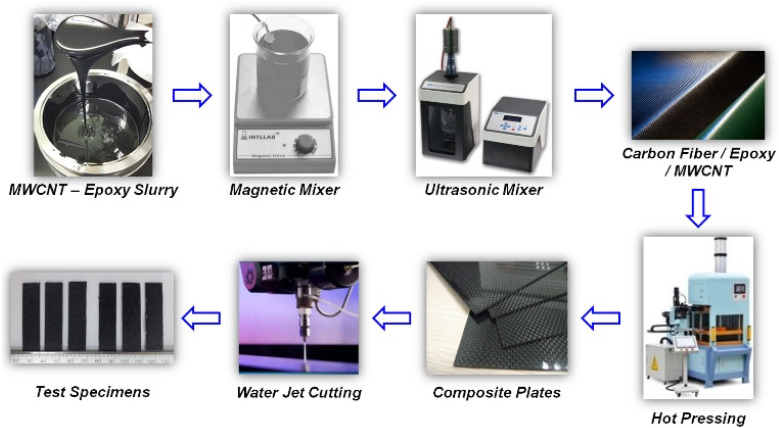
Schematic diagram of CFs/EP/MWCNTs composite bipolar plate preparation.^63^

Darıcık F. *et al.*^63^ prepared composite bipolar plates modified with CFs/EP using MWCNTs, and in order to improve the lack of electrical conductivity of CFs/EP composite bipolar plates, MWCNTs were added for expanding the electrical conductive pathway of the composite bipolar plates as shown in Fig. 7. By reasonably controlling the amount of MWCNTs added, the electrical conductivity can be enhanced to a value of 120 S cm^−1^, which meets the DOE requirements. The addition of MWCNTs can also improve the mechanical properties, but the authors also found that an excessive amount of MWCNTs leads to a decrease in the mechanical properties due to the agglomeration of MWCNTs, and thus, it is necessary to control the amount of MWCNTs added to be under the agglomeration threshold value.

Similarly Kypta C. J. *et al.*^93^ prepared MWCNTs/nylon-6,6/CF composite bipolar plates using an injection molding method suitable for high-volume preparation of composite bipolar plates. The composite bipolar plates achieved electrical conductivity close to the DOE requirement (64 S cm^−1^) due to the fact that the MWCNTs could act as a conductive bridge between the CFs and were sufficiently dispersed during the injection molding process. Undoubtedly, this is propitious for the mass preparation of composite bipolar plates. In order to study the problem of MWCNTs agglomeration, Bairan A. *et al.*^94^ prepared composite bipolar plates by choosing different loadings of MWCNTs and found that the highest conductivity (158.32 S cm^−1^) could be achieved within the system when the CNTs loadings were 6%. At the same time, the highest mechanical properties were achieved slightly lower at a 5% mass fraction. Therefore, when using MWCNTs as conductive fillers, it is necessary to consider that MWCNTs may exhibit different thresholds for different properties.

**Fig. 8:**
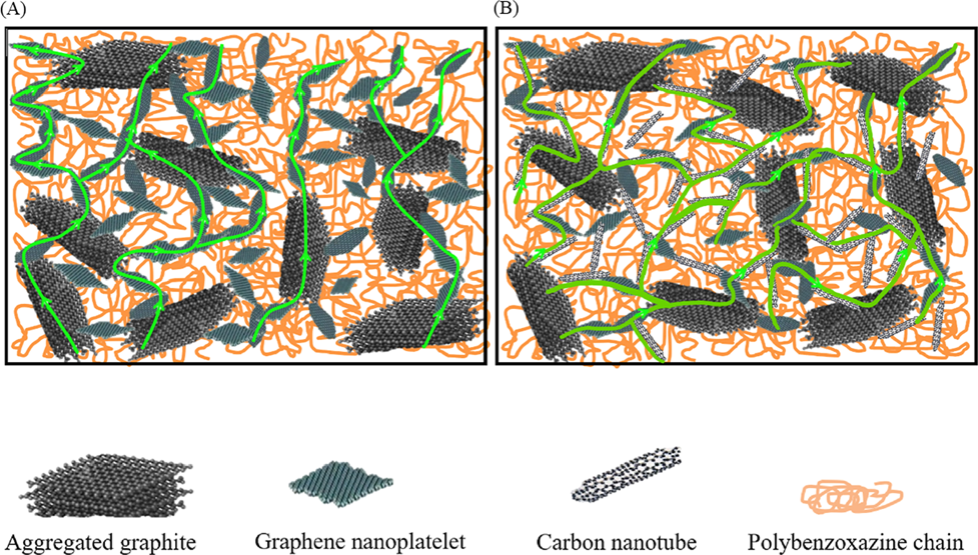
Schematic of thermal conduction path in (A) G/graphene filled PBA composite bipolar plates.^96^ (B) G/graphene/CNT/PBA of this research showing a greater continuous path of the composites than the composite without CNTs resulting in a much greater thermal conductivity.^60^

Witpathomwong S.^60^*et al.* prepared a new type of polybenzoxazine/multi-walled carbon nanotube/graphite composite bipolar plate with graphene added. Due to the high aspect ratio and high modulus of multi-walled carbon nanotubes^85^, and high strength improve the mechanical properties of the bipolar plate and extend the conductive path inside the bipolar plate, as shown in Fig. 8,^60,96^ the composite bipolar plates showed goo d performance with a planar conductivity of up to 364 S cm^−1^ and flexural strength of 41.5 MPa. This research indicates that the composites prepared with nanofillers are one of the best candidates for bipolar plates.

**Fig. 9:**
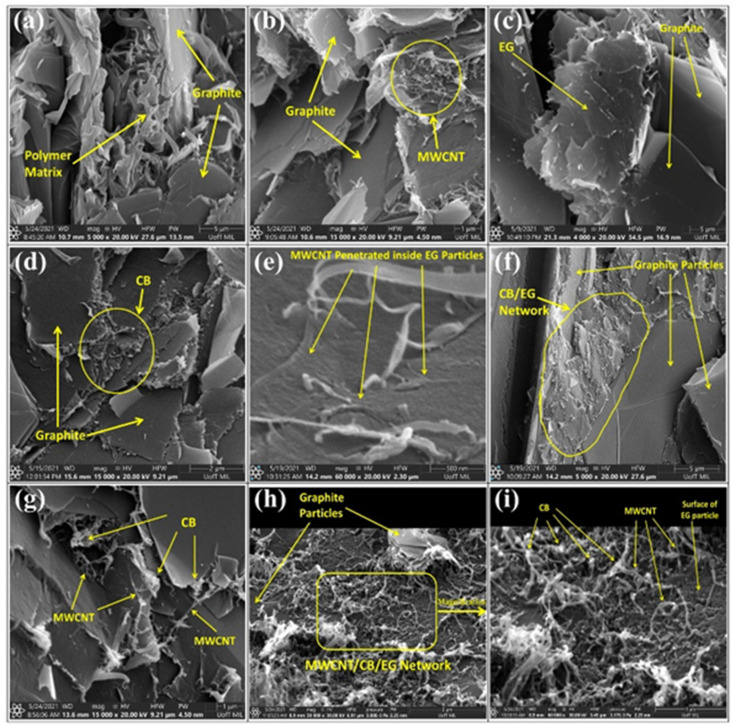
SEM micrographs of PP composites: (a) PP/G, (b) PP/G/MWCNT, (c) PP/graphite/EG, (d) PP/G/CB, (e) PP/G/MWCNT/EG, (f) PP/G/EG/CB, (g) P/G/MWCNT/CB, (h) PP/G/MWCNT/G/CB composites, (i) magnified view of composites bipolar plates.^80^

Tariq M.^80^*et al.* investigated the effect of the addition of carbon nanotubes/conductive carbon black on the properties of composite bipolar plates, the aggregation of the morphology of the nanofillers after the addition was investigated, as shown in Fig. 9, and the effect of the addition of MWCNTs and conductive carbon black on the mechanical properties of the composite bipolar plates was investigated in detail by RSM modeling technique. The results show that MWCNTs and carbon black have a significant effect on the mechanical properties, and the nanofiller is the factor that significantly affects the properties of composite bipolar plates compared to graphite matrix. By rationally configuring the raw material ratios, the composite bipolar plates exhibited a planar conductivity of 39.6 S cm^−1^ and flexural strength of 29.4 MPa at the raw material ratios of 4 wt% MWCNTs, 5 wt% CB, and 30 wt% EG with 64% PP.

MWCNTs, as a composite material with high research fervor, have been tried for many applications in bipolar plates. Most composite bipolar plates have improved properties such as electrical conductivity and flexural strength. Although MWCNTs show excellent properties, most reports also point out that MWCNTs can agglomerate in case of excessive additions, leading to the degradation of properties. Therefore, the amount of MWCNTs used should be carefully considered. In the future, to fully utilize the potential of MWCNTs as nano-fillers, it is also worthwhile to investigate how to rationally disperse and improve the agglomeration phenomenon of MWCNTs.

### 2.3 Carbon nanofibers

Carbon nanofibers (CNFs) have a similar morphology to carbon nanotubes but have different properties. CNFs have a lower crystallinity than CNTs, and some CNFs have larger diameters and poorer mechanical properties. However, CNFs are simpler to prepare, more versatile, and relatively low-cost; therefore, CNFs are often considered one of the candidate nanofillers for composite bipolar plates.

**Fig. 10:**
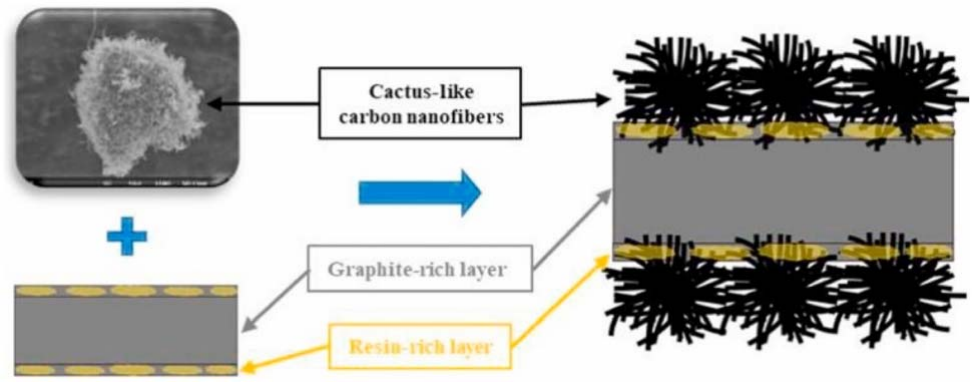
Schematic morphology of CNFs-modified composite bipolar plates.^55^

Jiang F. *et al.*^55^ prepared a cactus-like CNFs-modified graphite, as shown in Fig. 10, and synthesized a composite bipolar plate with EG and PVDF on this basis. CNFs can not only effectively improve the conductive pathway inside the composite bipolar plate by itself and expand the contact channels between the conductive particles but also destroy the resin-rich layer on the surface where the resin has been extruded due to the molding process. The CNFs are also used in the composite bipolar plate to improve the conductive pathway. This reduces the contact resistance of the composite bipolar plate. From the results, the CNFs-modified composite bipolar plates exhibit a planar conductivity of 198.7 S cm^−1^ and an area-specific resistance of 25.4 mΩ cm^2^; in addition, the graphitization degree of the graphite phase of the CNFs-modified composite bipolar plates increases, so the electrochemical stability is more robust.

Ramírez-Herrera C. A. *et al.*^59^ also conducted a study for composite bipolar plates modified with CNFs. The performance differences between CNFs and MWCNTs were compared, and their possible synergistic effects were also investigated. The results showed that CNFs alone did not enhance the strength as much as MWCNTs. However, the addition of CNFs to hybrid nanocomposites using both of them could effectively improve the performance of composite bipolar plates, which may be since both CNFs and MWCNTs can participate in the construction of the conductive network. With 15% CNFs and CNTs, the composite bipolar plates achieved a flexural strength of 45.3 MPa and a planar conductivity of 8.2 S cm^−1^. Chris Calebrese^100^ investigated the potential of CNFs-modified intermediate-phase bitumen as a composite bipolar plate material, which was able to slow down swelling due to the intertwined structure of CNFs that facilitates the reduction of the swelling effect of intermediate-phase bitumen due to the outgassing caused by the heating and carbonization process. The results show that the composites exhibit good electrical conductivity (40–80 S cm^−1^) and flexural strength (40–0 MPa), which are potential alternative materials for composite bipolar plates.

Overall, CNFs as a kind of nanofillers have a great impact on improving the electrical conductivity of composite bipolar plates, the large specific surface area of CNFs can perfect the conductive pathway, and the characteristics such as CNFs are easy to prepare compared with other nano-fillers, which make them also used as one of the competitive nano-fillers.

### 2.4 Graphene

Unlike MWCNTs and CNFs, which are one-dimensional, and CB, which is zero-dimensional, graphene (GNP) is a two-dimensional carbon nanoconductive filler. As a carbon-based nanofiller, graphene has good thermal and electrical conductivity as well as high intrinsic mechanical properties, which enables its application in composite bipolar plates^97^ because its large specific surface area can be involved in connecting different conductive regions and can itself serve as a stress-bearing point to enhance the mechanical properties.

Adloo A. *et al.*^77^ used GNP paired with CB to prepare PP/G composite bipolar plates. PP grafted with maleic anhydride (MAH) was used as a solubilizer to improve graphene dispersion. The graphene flakes were better dispersed in the matrix, and thus, the various properties of the composites were improved. The flexural strength of the composite bipolar plates could be increased from 43.83 MPa to 51.51 MPa with the addition of only 1% graphene, and the increase in the electrical conductivity was even more pronounced from 5.3 S cm^−1^ to 9.78 S cm^−1^. This suggests that the well-dispersed graphene can significantly enhance the performance of the composite bipolar plate; however, when the addition of graphene is 2%, the performance of the composite bipolar plate starts to decrease due to agglomeration instead. Therefore, the amount of graphene used needs to be carefully selected. Radzuan N. A. M. *et al.*^76^ investigated the preparation of composite bipolar plates using milled carbon fibers (MCFs), MWCNTs, and graphene nanosheets. They compared the effect of MWCNTs and graphene nanosheets on the properties due to graphene nanosheets filling the pores, which facilitates the formation of a complete electrical conductive pathway. The results showed that the electrical conductivity of composite bipolar plates can reach 3.42 S cm^−1^.

**Fig. 11:**
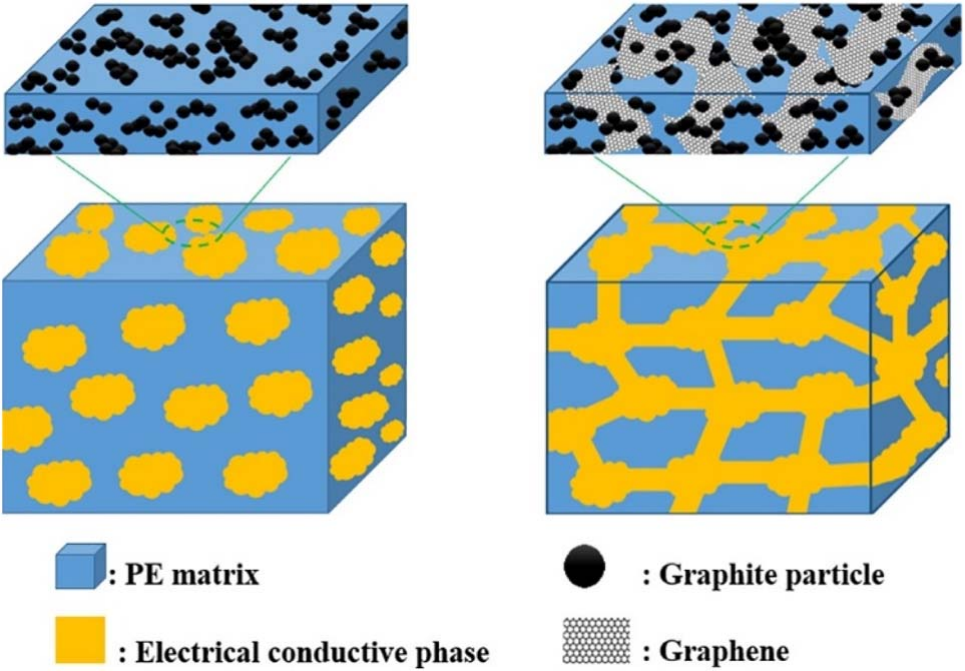
Schematic diagram of graphene and graphite constructing the conductive pathway.^98^

Liao W. *et al.*^98^ investigated composite bipolar plates using graphene/G/CF ternary conductive filler with polyethylene. Through the high conductivity of graphene, the composite bipolar plates were able to achieve high electrical conductivity with low carbon content, and the area resistivity and in-plane conductivity were able to satisfy the DOE requirements, which were 5.0 mΩ cm^2^ and 420.6 S cm^−1^, respectively. The network structure of the composites was formed between the graphene, graphite, and CF, as shown in Fig. 11, so only a tiny addition amount (25 wt%) is needed to achieve good electrical conductivity. In contrast, a higher resin addition amount improves the mechanical properties and corrosion resistance of the composite bipolar plates.

**Fig. 12:**
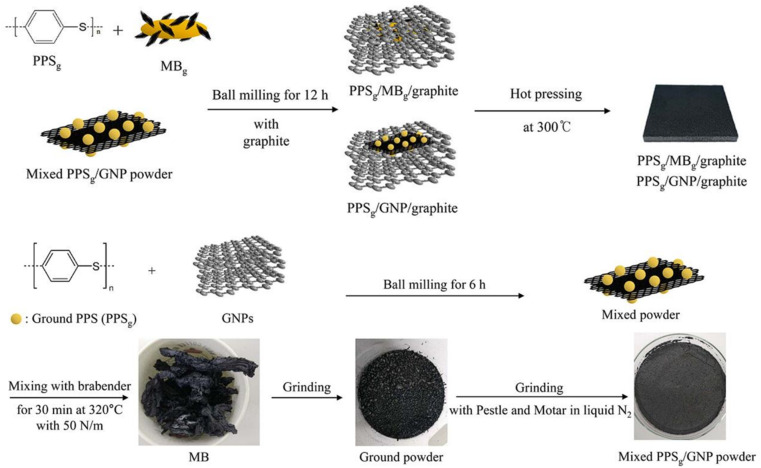
Schematic diagram of the preparation of PPS/G/GNP composite bipolar plate.^99^

To improve the dispersion of GNP in graphite/resin matrices, Kim S. H.^99^ prepared composite bipolar plates using polyphenylene sulfide (PPS) and graphite and investigated the effect of the addition of GNP on the performance of composite bipolar plates. In order to fully utilize the performance of GNP, the composite bipolar plates were firstly prepared by mixing GNP with PPS and subsequently with graphite as well as more PPS, as shown in Fig. 12. Due to the multi-step dispersion, the GNP was well dispersed in the matrix, and no agglomeration was still observed at 5% GNP addition, and the electrical conductivity of the composite bipolar plates increased from 643 S cm^−1^ to 1340 S cm^−1^, and the thermal conductivity was also improved by more than 100%. However, the authors also pointed out that too much graphene also led to a slight decrease in the mechanical properties.

**Fig. 13:**
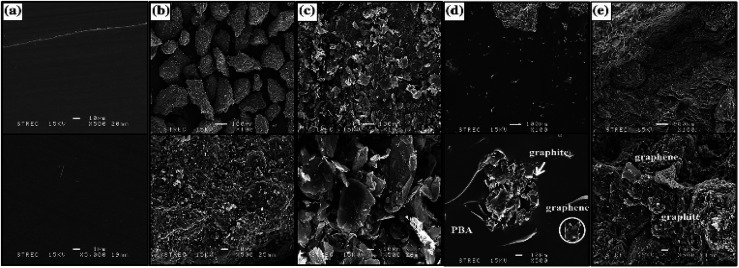
SEM micrographs of fracture surface of graphite-/graphene-filled PBA composites: (a) the neat PBA matrix, (b) pure graphite, (c) pure graphene, (d) 0.5/0.5 wt% graphite-/graphene-filled PBA, (e) and 80.5/2.5 wt% graphite-/graphene-filled PBA.^96^

Similarly, to improve the dispersion of GNP in composite BPs, Phangngamphan M. *et al.*^96^ prepared composite bipolar plates using GNP, PBA, and graphite and compared the effect of the addition of GNP on the performance of composite bipolar plates. By using the lower viscosity resin PBA, GNP was able to be well dispersed in the samples, resulting in more complete and uniform conductive pathways, the morphology of which is shown in Fig. 13. Replacing a small amount of graphite with GNP composite bipolar plates improved the conductivity compared to the control, from 291 S cm^−1^ at 2.5 wt% addition to 329 S cm^−1^ at 10% addition. However, the strength slightly decreased from 59 MPa to 54 MPa at 10% addition as the graphene loading increased, which was attributed to the increase in porosity due to the excessively high specific surface area of graphene.

In conclusion, graphene, as a widely used nanofiller, has made sufficient research progress in composite bipolar plates, and most of the research results have demonstrated that graphene can substantially improve the electrical conductivity of composite bipolar plates. However, the agglomeration problem of graphene due to its high surface energy and the high cost of the amount of graphene to be used needs to be carefully considered. Therefore, more consideration should be given to the agglomeration problem of graphene as well as the selection of suitable dispersion methods in future research. Overall, graphene composites are still one of the candidates for future bipolar plate materials.

### 2.5 Other nanofillers

In addition to carbon-based nanofillers, other types of nanofillers, such as copper nanoparticles,^101^ SiC nanoparticles,^102^ and metal oxide nanoparticles,^103^ have also been shown to be used as enhancers of the performance of composite bipolar plates. Non-carbon-based nanoparticles are mainly focused on enhancing the mechanical properties, and there are differences in the performance and the cost compared to carbon-based fillers.

Soleimani Alavijeh M.^101^*et al.* prepared composite bipolar plates using copper nanoparticles with epoxy resin–graphite. The well-distributed nanoparticles not only enhanced the mechanical properties of the composite bipolar plates but also improved their electrical conductivity. The electrical resistivity decreases rapidly after the addition of copper nanoparticles exceeds the threshold value, and the electrical resistivity decreases from 3% of the addition up to 5%; the electrical resistivity increases from 7400 mΩ cm^−1^ to 1200 mΩ cm^−1^. The flexural strength can also increase from 36.15 MPa to 40.34 Pa, which is due to the fact that the copper nanoparticles can fill the pores of the graphite and withstand more stress concentration.

**Fig. 14:**
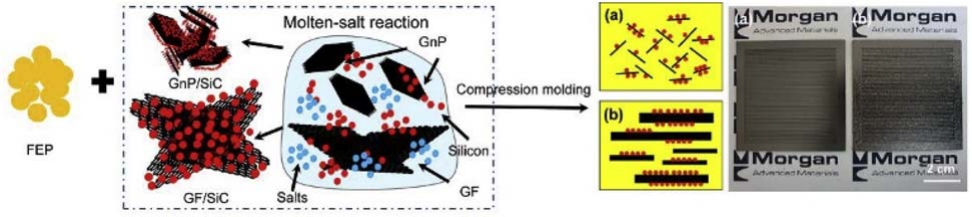
Schematic of the procedures for preparing FEP/GnP/SiC composite bipolar plates.^102^

Park *et al.*^102^ prepared composite bipolar plates using graphite nanosheets with nanoscale silicon carbide as well as graphite scales and FEP resin and investigated the effect of SiC in increasing stillness. As shown in Fig. 14, graphite nanosheets loaded with nano-SiC particles were obtained by mixing graphite nanosheets, Si particles, and CaCl. SiC particles can play a role in carrying stress concentration, and due to the homogeneous dispersion as well as the strong bonding with the matrix, the mechanical properties of the composites were greatly improved, and the flexural strength of the composite bipolar plates can be increased by up to 130%, which exceeds 50 MPa.

In addition, Soleimani Alavijeh^103^*et al.* also explored the possibility of metal oxide nanoparticles as nanofillers for composite bipolar plates and found that metal oxide particles have the potential to be used as nanofillers enhancers for composite bipolar plates by exploring the effect of using replacing some of the graphite with zinc oxide as well as titanium dioxide. From the results, it was found that the addition of titanium dioxide nanoparticles has a positive effect on the electrical conductivity within the appropriate range, while zinc oxide improves the flexural strength. At 6% ZnO addition, the flexural strength of composite bipolar plates can meet the DOE requirements for 2025 (>45 MPa).

In short, the development of novel other nanofillers has also been shown to be feasible as it helps to reduce the high cost of traditional carbon-based fillers; however, most of the results on non-carbon-based nanofillers have shown a relatively significant enhancement in the mechanical properties and a slight enhancement in the electrical conductivity, which may be attributed to the intrinsically lower electrical conductivity of the other nanofillers as compared to that of the carbon-based nanofillers, and hence how the bending strength can be enhanced by other nanofillers without compromising on the electrical conductivity is also a matter of concern.

### 2.6 Summary in nanofillers

At this stage, several nanofillers have been demonstrated to enhance the performance of composite bipolar plates, such as MWCNTs, CNFs, GNP, CB, and so on. Adding these fillers can effectively enhance the various properties of composite bipolar plates, thus realizing the practical use of composite bipolar plates. Although zero-dimensional CB, one-dimensional CNF, CNT and two-dimensional GNP can effectively improve the mechanical and electrical conductivity of composite bipolar plates, their focus and mechanism are different, and they need to be selected according to the actual production needs. Carbon black as a point filler, mainly through the filling of defects and pores to form a more perfect conductive pathway and material structure, and can be strengthened by diffusion to withstand the stress concentration to enhance the mechanical properties. One-dimensional CNF, CNT and two-dimensional GNP connect the conductive phases of the material with the help of their specific structure to form an overall conductive pathway. The properties of composite bipolar plates modified with various nanofillers are shown in Table 2.

**Table 2:** Performance of composite bipolar plates with added nanofillers

Materials	In-plane electrical conductivity S cm^−1^	Flexural strength	Reference
MWCNT/PP/G	548	>30	91
MWCNT/EP/NG	129	27.05	92
CF/EP/MWCNT	120	47	63
MWCNT/nylon/CF	64	—	93
MWCNT/CB/G/PP	158.32	29.86	94
Fenton-CF/EP	∼250	∼38	85
MWCNT/GNP/G/PBA	364	41.5	60
CB/CF/PF	—	48.6	95
CB/SG/PVDF			96
CB/EG/PVDF	177.87	49.16	89
EP/NFG/CB	169	43	64
MWCNT/CB/G/PP	49.25	34.8	66
GNP/CB/G/PP	9.78	51.51	77
GNP/MWCNT/MCF/PP	3.42	—	76
GNP/CF/G/PE	420.6	28.4	98
GNP/G/PPS	1340	26	99
GNP/G/PBA	329	54	94
CNF/EG/PVDF	198.7	—	55
MWCNT/CNF/G/?	8.2	45.3	59

However, in composite bipolar plates, nanofillers face some problems, such as easy to agglomerate and difficult to disperse,^63,66,77,91^ which increases the application cost of nanofillers. In contrast, agglomerated nanofillers have a negative impact on the performance of composite bipolar plates. Another concern is that most of the nanofillers have poor wettability with the resin, and over-addition may produce structures such as pores due to poor wettability,^96^ which in turn affects the overall performance of the composite bipolar plates. Therefore, in future research, in order to solve the above problems, it is worthwhile to select suitable nanofillers to match with resins or to improve the affinity of nanofillers with resins by changing the surface properties of nanofillers. In addition, the cost of nano-fillers is relatively high, so it is necessary to choose the amount of addition carefully, therefore, how to reduce the cost can also be effective to promote the practical application of composite bipolar plates. In conclusion, the application of nano-fillers in composite bipolar plates has developed rapidly in recent years, and nano-fillers have been proved to be one of the reliable methods to promote the practicality of composite bipolar plates, but the problems of nano-fillers being easy to be agglomerated, having poor wettability with resin, and having high cost need to be solved by more comprehensive research in the future.

## 3 Structural design and optimization

In addition to the addition of nanofillers, another major current research direction for composite bipolar plates is the optimization and design of the structure of composite bipolar plates. Structure optimization through reasonable methods can improve the performance of composite bipolar plates without changing the graphite/resin raw material ratio.

At present, the main directions of structural optimization are: (1) optimization of the dispersion pattern of graphite/resin,^104–107^*i.e.*, through physical or chemical means, artificially control the dispersion pattern between graphite/resin phases, so that the composite bipolar plate has a strategic distribution of graphite–resin, (2) optimization of the electrically conductive network of the composite bipolar plate:^108–110^ through the design of electrically conductive paths of the composite bipolar plate, so that the composite bipolar plate has the overall electrically conductive network structure. (3) Interfacial modification and surface functionalization of the fillers of the composite bipolar plate^111,112^ to enhance the bonding force between the interfaces of the composite bipolar plate and the wettability between the graphite–resin.

It should be noted that the optimization of the structure is not in conflict with the use of nanofillers, and thus, the combination of the two can lead to superior performance of the composite bipolar plates. In the following subsection, the research results of the structure optimization of composite bipolar plates in recent years, as well as the advantages and challenges, will be classified in detail.

### 3.1 Graphite/resin distribution optimization

For most composite bipolar plate preparation processes, the raw materials are often mixed mechanically and prepared into bipolar plates using molding. This will meet two common problems: (1) in the mechanical mixing process, graphite and resin tend to be concentrated rather than uniformly distributed due to poor wettability, which reduces the electrical conductivity and mechanical properties. (2) In the molding process, due to the better fluidity of the resin, it will lead to the composite bipolar plate surface of the resin is more enriched, while the middle of the resin is less, which will lead to the contact resistance rise. Therefore, it is necessary and feasible to control the dispersion between graphite/resin by artificial modulation to improve the above problems and enhance the performance of composite bipolar plates.

**Fig. 15:**
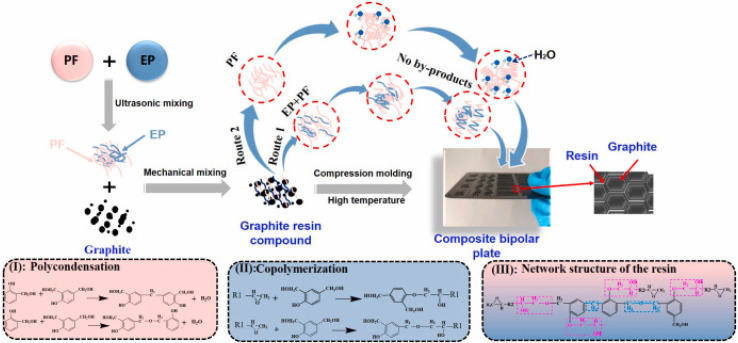
Schematic diagram of epoxy/phenolic resin copolymer and composite bipolar plate preparation.^113^

Chen J. *et al.*^113^ innovatively optimized the resin network structure in composite bipolar plates by copolymerizing the resin. As shown in Fig. 15, by copolymerizing the epoxy resin with phenolic resin, the resin is made to have both hydroxyl and flexible chain segments, which improves the adhesion between the resin and graphite greatly and facilitates the mutual dispersion of the two. The composite bipolar plate exhibited a composite DOE-required flexural strength of 46.2 MPa as well as a planar conductivity of 188 S cm^−1^ and a power density of 534.76 mW cm^−2^, and this performance enhancement was not at the expense of changing the material ratios as well as adding new fillers, which makes it potentially suitable for large-scale applications.

**Fig. 16:**
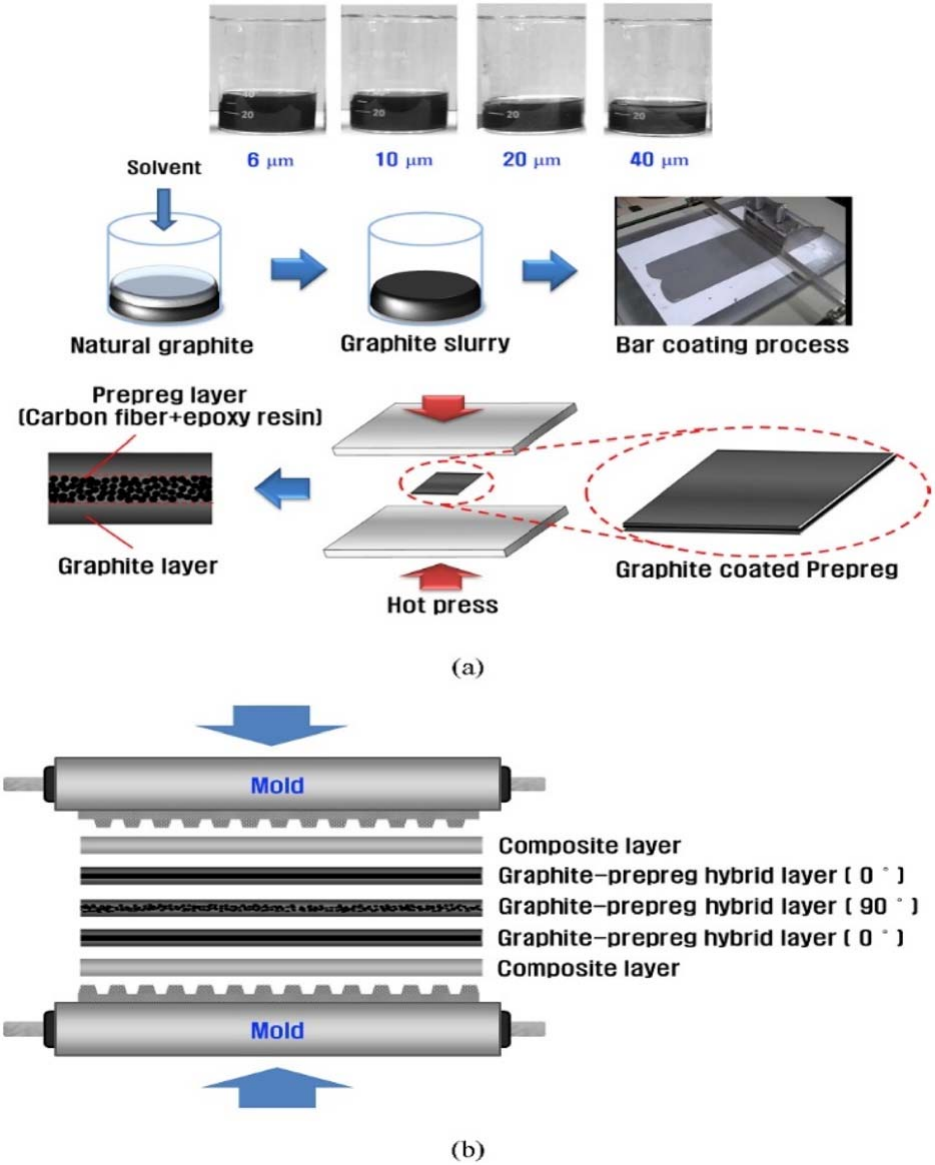
(a) Schematic of the fabrication procedures for the composite coated graphite–prepreg hybrid BPs and (b) compression molding process.^47^

**Fig. 17:**
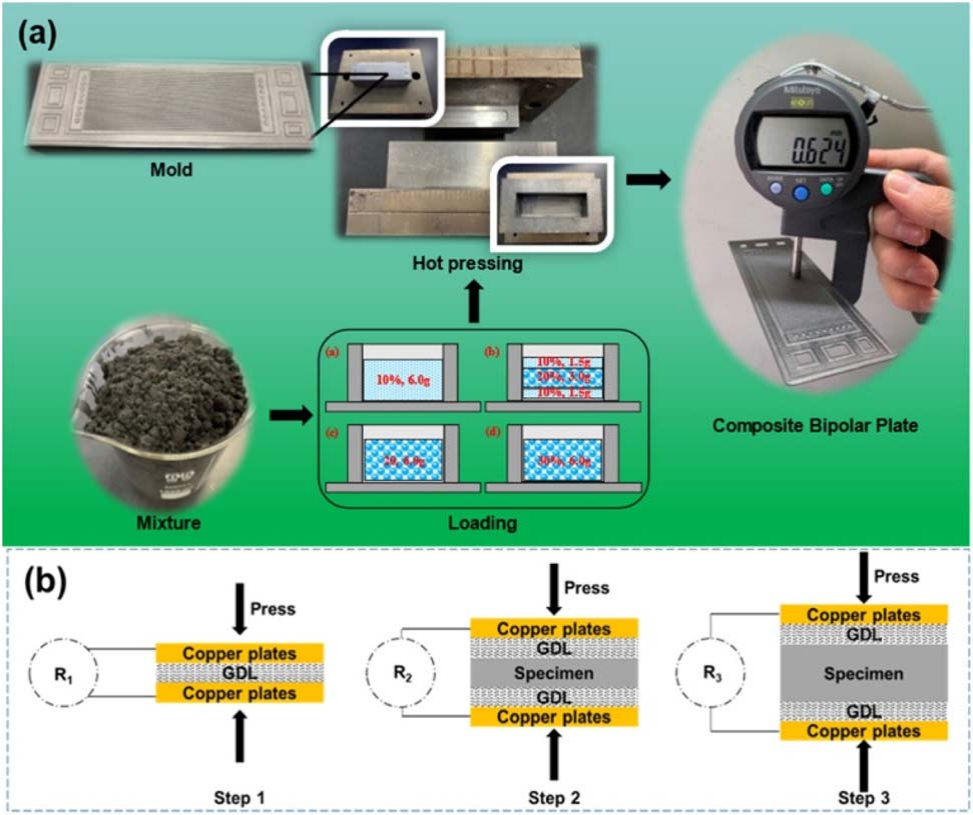
(a) Schematic diagram of laminated structure composite bipolar plate preparation, (b) schematic diagram of contact resistance detection.^106^

In order to solve the phenomenon of resin enrichment on the surface due to resin being extruded during the molding process of composite bipolar plates, Kang K. *et al.*^47^ designed a composite bipolar plate with a laminated structure, as shown in Fig. 16, in which a pure graphite layer without resin is inserted into the bipolar plate to absorb the extruded resin, thus improving the distribution of the resin/graphite in the thickness direction, and it is no longer the case that the resin of conventional molding is on the surface and is enriched at the surface and lacking in the middle of the morphology of conventional molding. At the same time, the graphite/resin composite layer is laid on the surface in the form of a thin layer, which contains a certain amount of resin and is easy to mold in the flow channel. By optimizing this laminated structure, the composite bipolar plate has a good conductivity of 172 S cm^−1^. Similarly, Zhang X.^106^*et al.* prepared a laminated composite bipolar plate, as shown in Fig. 17, with a resin content of 10% in the surface prefabricated layer and 20% in the intermediate layer, such that the resin flowed from the high-resin area in the intermediate to the low-resin area during molding, which led to a more balanced resin distribution in the composite bipolar plate. As the resin-enriched distribution on the surface is effectively avoided through suitable structural optimization, the electrical conductivity of the composite bipolar plate is effectively enhanced, the surface contact resistance is reduced to 9.2 mΩ cm^2^ @ 100 psi, and a flexural strength of 44.3 MPa is demonstrated due to the uniform distribution of the resin to avoid the concentration of stresses at the vulnerable points.

**Fig. 18:**
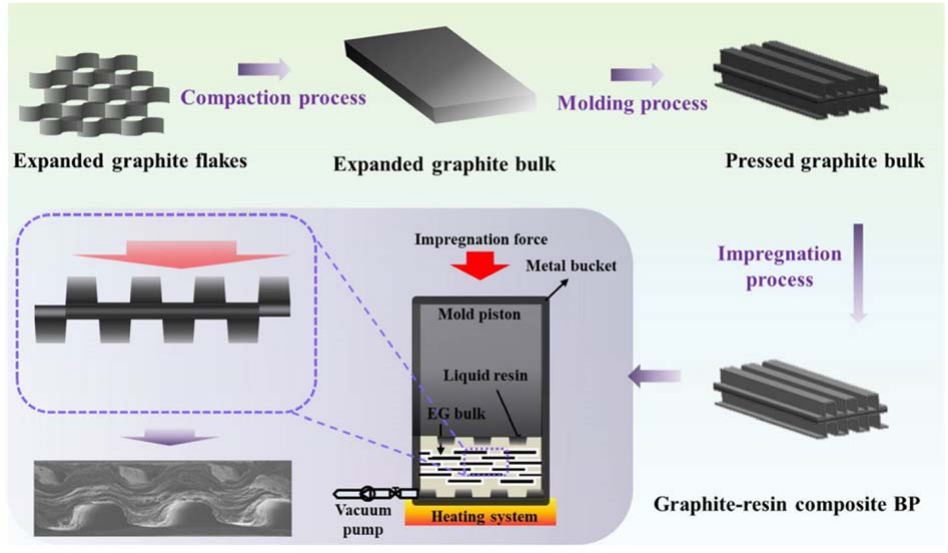
Preparation process for impregnated/molded composite bipolar plates.^114^

Wang X.^114^*et al.* prepared composite bipolar plates by vacuum impregnation method using expanded graphite as raw material, as shown in Fig. 18 and found that the tendency of the resin was distributed in the top regions of the rib under high impregnation pressure. By modulating the molding and impregnation pressures, the distribution pattern of the resin can be effectively controlled to enhance the performance of the composite bipolar plates. By concentrating the resin distribution in the top regions of the rib, the additivity of the runner region, which requires high processability, is enhanced, while the main region of the composite bipolar plate retains the complete graphite network structure, which is conducive to the rise of electrical and thermal conductivities, and its in-plane conductivity can reach 400 S cm^−1^.

In addition to modification by chemical methods, preparation of laminates, and optimization of process parameters, it is also beneficial to improve the performance by adjusting the particle size of the raw material for composite bipolar plates. Z. Jie^115^*et al.* investigated the effect of expanded graphite particles on the performance of composite bipolar plates and found that smaller graphite particles can enhance the mechanical properties but are not conducive to the connection of the conductive pathways, and the conductivity decreases, whereas larger graphite particles, on the contrary. Chunhui S.^116^*et al.* prepared composites using graphite with different particle sizes, which made the connection of graphite conductive phases closer by filling the gaps between large particles with small particles, thus effectively enhancing the electrical conductivity.

In summary, there are many ways to change the distribution of graphite/resin to optimize the structure, mainly (1) by physically/chemically changing the wettability between the resin and graphite, (2) by pre-fabricating preforms containing different resin mass fractions and then molding them to promote uniform distribution of the resin, (3) by optimizing the process parameters in the molding process to regulate the distribution of the resin/graphite, and (4) through the strategically using particles with different particle sizes so that smaller particles can fill in the voids of larger particles, resulting in fewer voids and a more holistic distribution of the conductive phase. By optimizing the graphite/resin distribution pattern to enhance the performance of composite bipolar plates, neither additional conductive filler is needed, and it is also simpler to implement in the actual production, which is very conducive to the high performance and practicality of composite bipolar plates.

### 3.2 Conductive pathway optimization

In addition to artificially regulating the phase distribution of graphite/resin to enhance performance, it is also feasible to optimize and design the conductive pathways inside the composite bipolar plates. For most of the composite bipolar plate, the distribution of conductive particles is random, and it is difficult to connect with each other to form a complete conductive pathway; if the conductive pathway is connected to each other as a whole, it will make the conductivity of the composite bipolar plate rises dramatically. A number of methods are currently used to optimise the conductive pathways: for example the use of porous carbon preforms followed by diafiltration of the resin to form bipolar plates with integral conductive pathways, or controlling the morphology of the conductive pathways by adjusting the morphology of the conductive filler.

**Fig. 19:**
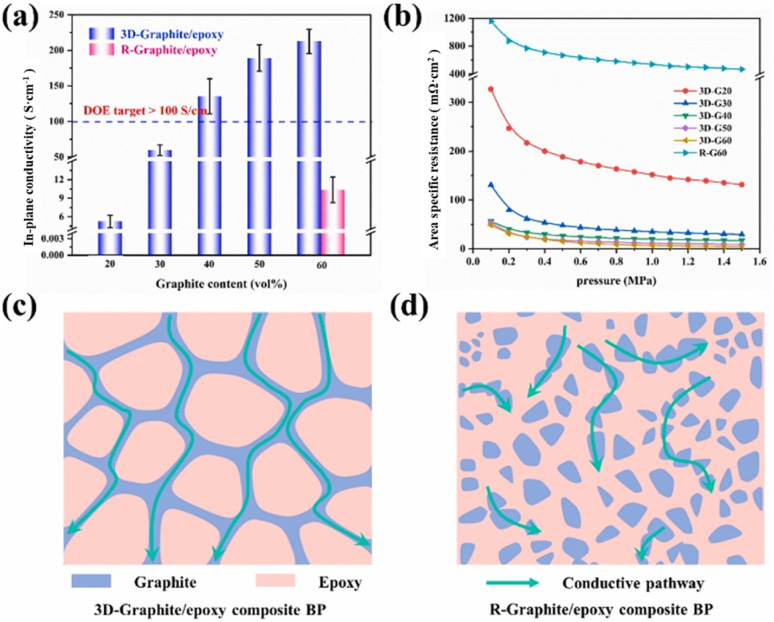
(a) In-plane conductivity and (b) ASR of the 3D-graphite/epoxy composite BP, the schematic diagrams of conductive pathway: (c) composite bipolar plates with selective distribution. (d) Composite bipolar plates with random distribution of graphite.^117^

Hu B. *et al.*^117^ used a sacrificial material to prepare a composite bipolar plate with a three-dimensional continuous conductive network, which improved the conductivity and maximum power density compared to the composite bipolar plate without strategically designed conductive pathways. A porous 3D graphite skeleton was prepared by mixing NH_4_HCO_3_ with graphite and then heating to remove the NH_4_HCO_3_, and then the composite bipolar plate was prepared by filling the graphite skeleton with resin through impregnation, which resulted in the formation of a 3D network of conductive pathways in graphite as shown in Fig. 19. Thanks to this mechanism, the highest in-plane conductivity of the composite bipolar plate is up to 212.64 S cm^−1^, and the power density is 317.52% higher than that of the composite bipolar plate prepared by the traditional method, up to 853.42 mW cm^−2^.

**Fig. 20:**
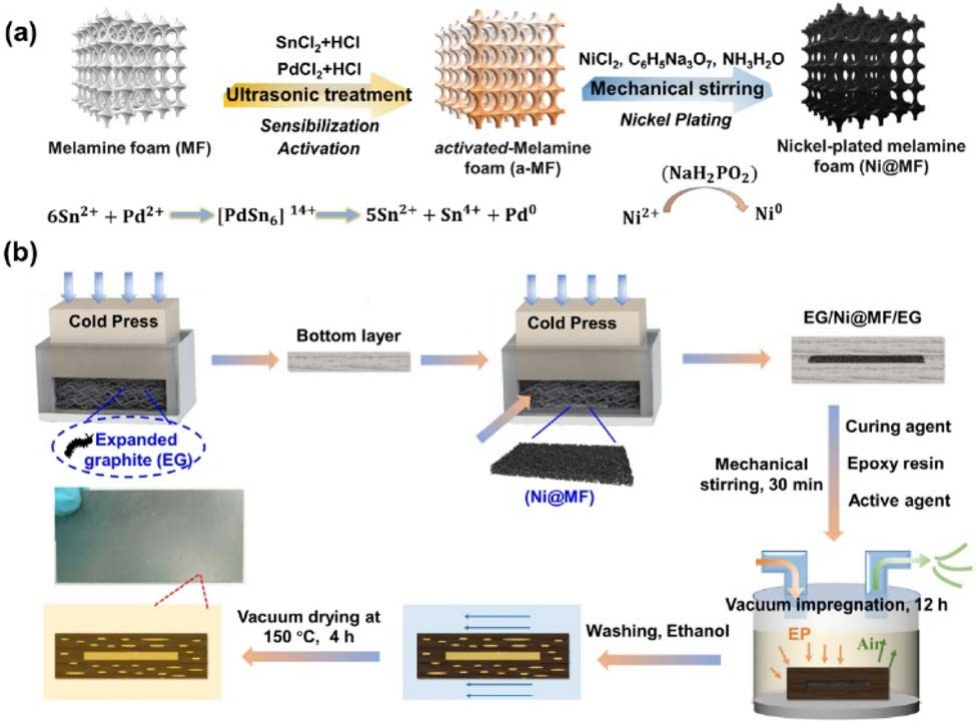
(a) Schematic diagram for the preparation of Ni@MF; (b) preparation process of EG/Ni@MF/EG–EP composite bipolar plate.^68^

Mao *et al.*^68^ used the method shown in Fig. 20 to prepare Ni@MF sponges by chemical plating using melamine foam (MF) as a substrate and laminating it with graphite to obtain an EG/Ni@MF/EG prefabricated plate, followed by the preparation of composite bipolar plates using an epoxy resin impregnation process. As the plated nickel relies on the three-dimensional MF skeleton to form a complete and continuous three-dimensional structure, which provides a channel for electron transport, and the porous MF frame provides enough space for resin impregnation, the conductivity of the composite bipolar plates was increased to 320 S cm^−1^. The filled resin also improves the mechanical properties of the composite bipolar plates. Therefore, the composite bipolar plates also show good flexural strength and airtightness, with flexural strength. The composite bipolar plate also showed good flexural strength and airtightness, with a flexural strength of 56 MPa, and other properties that meet the requirements of the 2025 DOE.

**Fig. 21:**
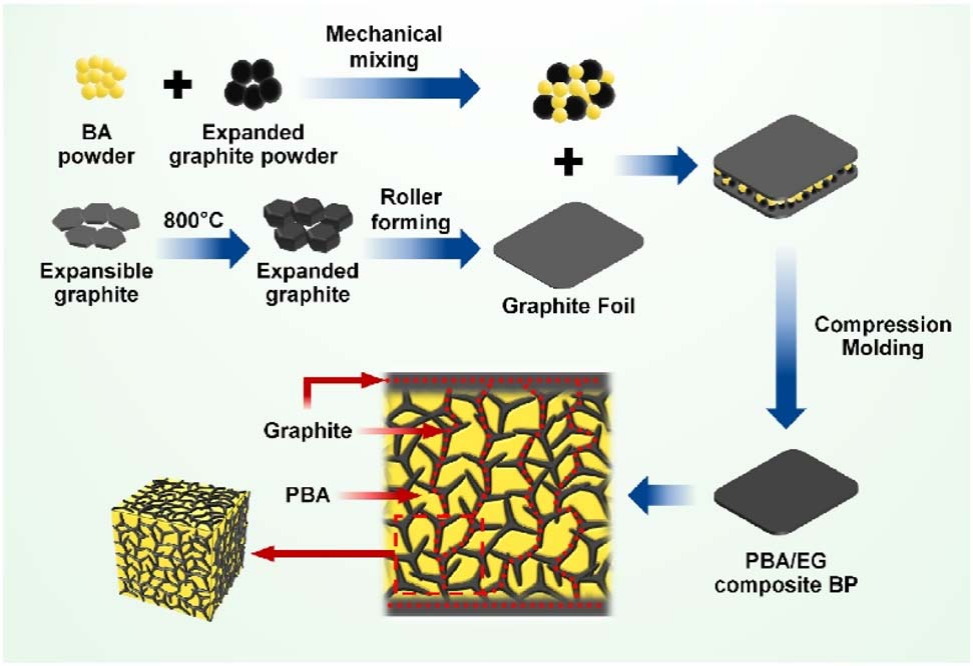
Schematic of the preparation of PBA/EG composite bipolar plates.^108^

Huang Q.^108^*et al.* prepared composite bipolar plates with a conductive network structure using PBA and EG. Thanks to PBA's excellent fluidity and low viscosity, it can effectively flow during the molding process so that the conductive graphite can be connected. A cross-linking network structure was obtained as shown in Fig. 21, and at the same time, the graphite layer on the surface also prevents the resin from being enriched. Thanks to the strategically designed conductive network structure, the composite bipolar plate shows good performance with a conductivity of 278.58 S cm^−1^ and a flexural strength of 75.75 MPa due to the entire resin flow.

**Fig. 22:**
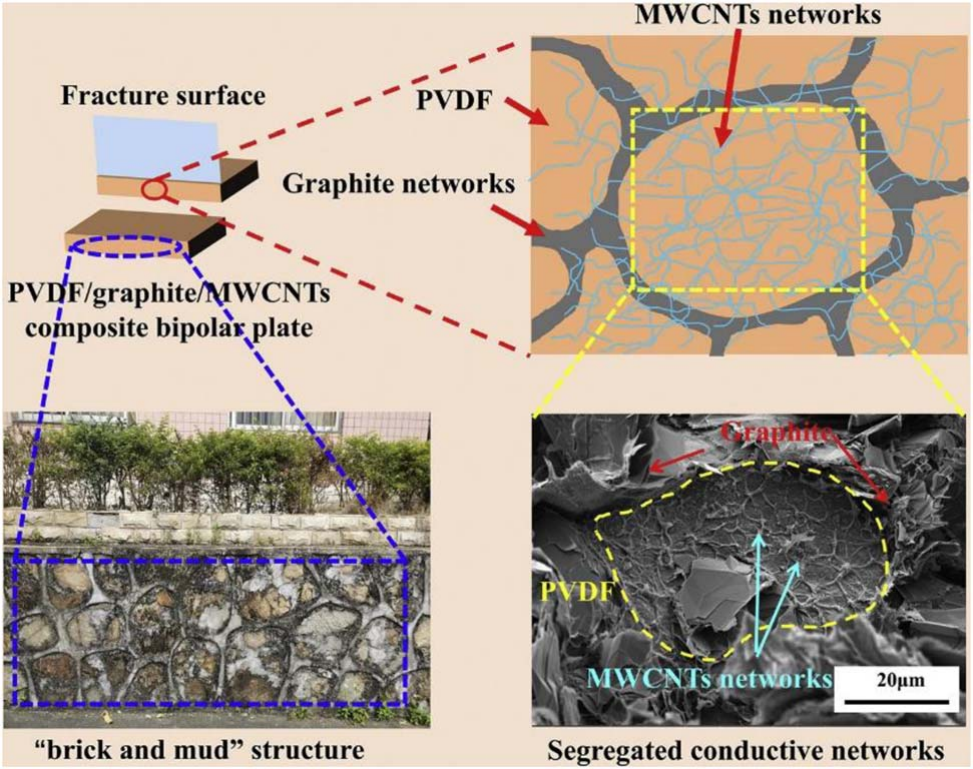
Schematic representation of graphite–MWCNTs segregated conductive networks.^109^

Similarly, Hu B.^109^*et al.* strategically mixed graphite with MWCNTs to obtain a mixed powder and then mixed it with resin to prepare a composite bipolar plate as Fig. 22 shows, and after mixing in a two-step approach, the graphite/MWCNTs produced a parabolic structure, which co-constructed a conductive network, forming a suitable conductive “brick-mud” structure. The selective distribution of MWCNTs and graphite broadened the conductive pathway, allowing the conductive particles to connect in three dimensions, so that the in-plane conductivity of the composite bipolar plate was increased to 161.57 S cm^−1^, and this structure also facilitated the MWCNTs to withstand more loads. Thus the flexural strength was improved to 42.65 MPa.

It is also feasible to improve the performance of composite bipolar plates by optimizing the structure of the conductive network, and in the last three years, this approach has attracted enough research heat. At present, composite bipolar plates with three-dimensional continuous conductive networks are mainly obtained by constructing the conductive network skeleton and then vacuum impregnation of resin, and it is also feasible to use the resin with good fluidity for dispersion to make the resin/graphite form a continuous interspersed structure. All in all, as a new type of performance enhancement method, the composite bipolar plate application shows a broad application prospect.

### 3.3 Interface structure optimization

During the preparation of composite bipolar plates, the poor interfacial bonding between graphite/resin/other fillers will result in internal voids and agglomeration of particles, which will lead to a decline in the performance of the composite bipolar plates, so the modification of the surface of the composite bipolar plate particles to improve the interfacial bonding is also one of the feasible means of structural optimization at present. For example, improving the wettability between fillers by functionalizing the surface of graphite/resin or enhancing the interfacial bonding by making the fillers selectively distributed at the interface is feasible.

**Fig. 23:**
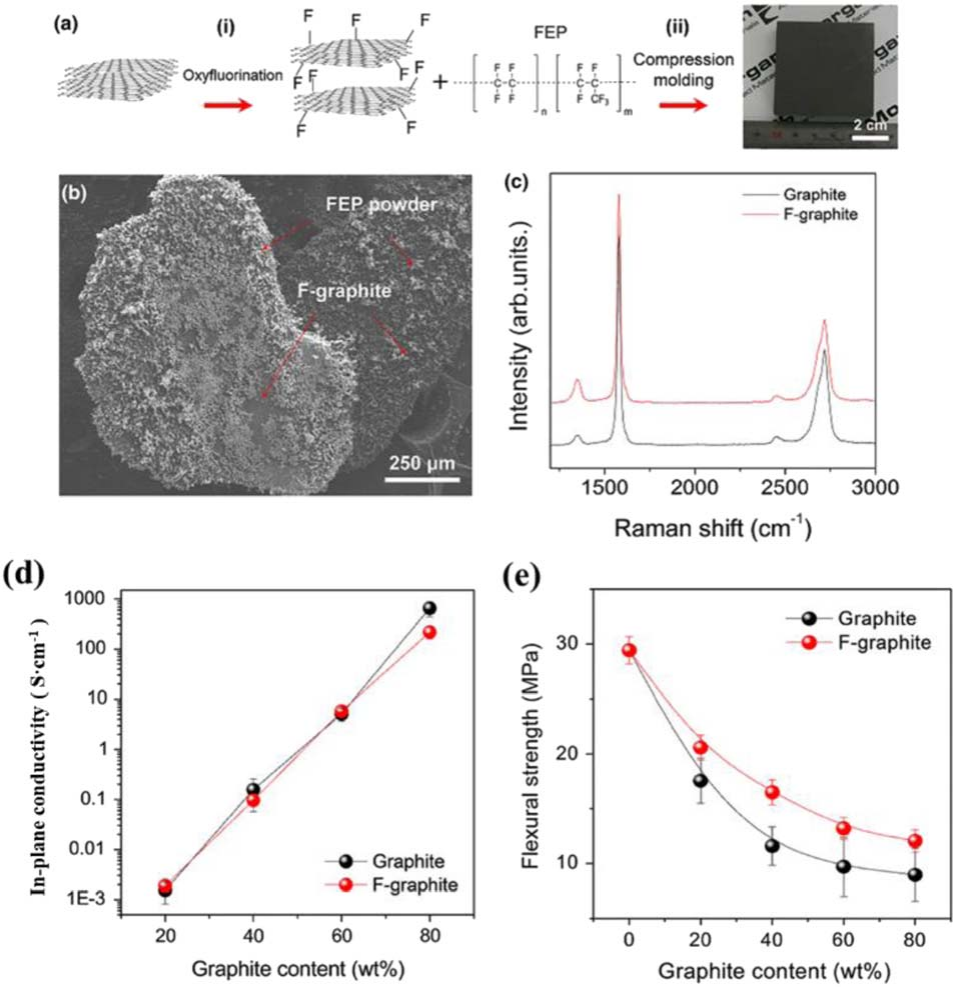
(a) Preparation and structure, (b) SEM, (c) XRD of fluorinated graphite/FEP composite bipolar plate. (d) In-plane conductivity. (e) Flexural strength.^111^

Lee M. H.^111^*et al.* prepared composite bipolar plates using functionalized fluorinated graphite as well as fluorinated ethylene–propylene (FEP). Compared with ordinary graphite, fluorinated graphite, and FEP showed higher interfacial compatibility and closer bonding between the interfaces, and the results in Fig. 23 showed that the FEP powder was better dispersed on the graphite matrix. Thanks to the tighter interfacial bonding can effectively increase the conductive pathway, although the intrinsic conductivity of fluorinated graphite is lower, but the electrical conductivity of the composite bipolar plate does not appear to be a significant decline, as Fig. 23 shows, while the mechanical properties are improved significantly.

Lv Bo *et al.*^85^ used modified carbon fibers as reinforcement for composite bipolar plates to improve the performance of the composite bipolar plates, in which the surface of the carbon fibers was treated with a Fenton reagent to improve the compatibility with the resin. Unlike the direct addition of carbon fibers, the fracture mechanism of the carbon fibers treated with two hours of Fenton reagent is different compared to that of ordinary carbon fiber/phenolic resin bipolar plates due to better bonding with the resin: without treatment, the carbon fibers tend to fail by being pulled out of the resin because of poor interfacial compatibility, and after the adhesion is improved by the surface treatment, the mechanical properties of the carbon fibers are fully utilized, which are involved in the stress-bearing during the fracture process. The treated carbon fiber-modified composite bipolar plate proton exchange membrane fuel cell has a maximum power density of 662.75 mW cm^−2^, which is valuable for practical application. It is worth noting that the increase in conductivity is closely related to the time of treatment of CFs with Fenton's reagent, which exhibits an increase in conductivity at lower treatment times due to the fact that the enhancement of conductivity by the improvement of the binding force is stronger than the decrease of the intrinsic conductivity of CFs by oxidation, whereas the opposite is true at higher treatment times.

Liao S. H. *et al.*^118^ used maleic anhydride for surface functionalization of MWCNTs, which led to the formation of copolymers of MWCNTs with poly(oxyethylene)amine (POA) of maleic anhydride (MA) through amide reaction. The surface functionalization improved the degree of dispersion of MWCNTs in the resin matrix, which effectively improved the mechanical properties and electrical conductivity of the composite bipolar plates. The excellent dispersion of MWCNTs led to better contacting of CNTs, and the in-plane conductivity could be enhanced to 643 S cm^−1^, while the good interfacial bonding also improved the mechanical properties of the bipolar plates to 41.44 MPa.

Similarly, Athmouni N. *et al.*^119^ studied the effect of functional group-functionalized MWCNT on the performance of composite bipolar plates, and they prepared MWCNT-COOH/PBT composite bipolar plates through nitric acid oxidation with MWCNTs and pre-blending MWCNT-COOH with polybutylene terephthalate (PBT). Due to the enhanced van der Waals force, the interface bonding between MWCNTs and PBT resin is greatly enhanced, resulting in a tighter interface bonding between resin and MWCNTs, effectively improving the mechanical properties. The results show that the bending strength of the composite bipolar plate with PBT-MWCNTs exceeds 12 MPa, which is about 50% higher than the control group with pure MWCNTs.

Methods similarly, Chaiwan P.^120^ used a strong acid to prepare functionalized MWCNTs and composite bipolar plates were prepared by improving the interfacial compatibility between MWCNTs and graphite/resin through silanization with a silane coupling agent. The results showed that thanks to the improved interfacial compatibility, the MWCNTs were better bonded to the substrate, and the mechanical properties rose, but the intrinsic electrical conductivity of the MWCNTs was reduced because of the disrupted structure of the MWCNTs, and the reduced graphitization, which consequently reduced the intrinsic electrical conductivity of the MWCNTs, but the electrical conductivity of the composite bipolar plates could still satisfy the DOE requirements.

**Fig. 24:**
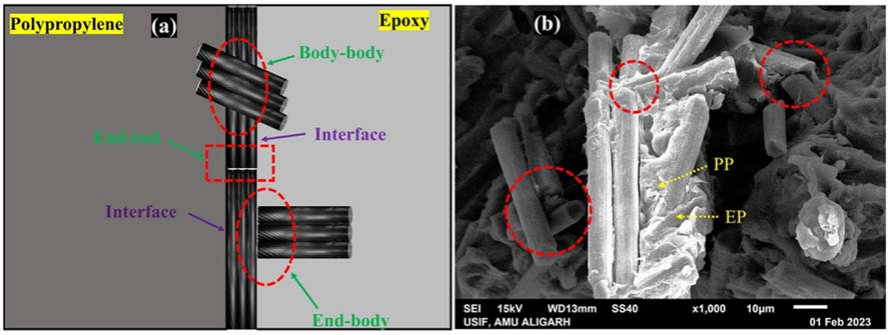
Three diverse patterns of contact between fibers in the PP/epoxy/CF polymer blend composites (PBCs): (a) diagrammatic illustration (b) SEM image.^121^

Al-Mufti S. M. S.^121^*et al.* prepared a composite bipolar plate using CFs as well as EP/PP copolymer blend (PB). Due to the use of two resins and the strategic use of two fillers, the CFs were modulated to selectively localize at the interface through thermodynamic and kinetic factors, as Fig. 24 shows. The head-to-tail oriented distribution is more favorable to enhance the performance of composite bipolar plates than the random distribution. The interfacially selectively distributed CFs, on the other hand, not only have a large contact area with the resin and a more stable attachment but also are more conducive to withstand large loads, and their flexural strength is the highest at 30% CFs addition, which is 72.72 MPa. Athmouni N. *et al.*^122^ improved the compatibility of MWCNTs with resin by functionalizing the surface of MWCNTs with nitric acid. The dispersion of MWCNTs in the resin matrix was improved by the formation of carboxyl groups on the surface of MWCNTs, and their planar conductivity was enhanced by 250%.

In summary, surface modification is mainly achieved by functionalizing the surface of graphite/other fillers by treating the surface with potent oxidizing agents and other reagents to bring on carboxyl or hydroxyl groups to improve the interfacial bonding with the resin. This strong interfacial bonding not only enhances the mechanical properties of the composite bipolar plates but also reduces the porosity, resulting in a tighter connection of the conductive regions and, hence, an improved conductivity. Surface modification can be categorized into surface modification of carbon-based fillers/resins according to the object of treatment. It has been reported that modification of the resin to promote interfacial bonding is often beneficial to the conductivity, but improving interfacial bonding by functionalizing the carbon-based filler is more sensitive to the process parameters, as functionalization tends to impair intrinsic conductivity, and therefore a balance needs to be found.

### 3.4 Summary

In conclusion, structure optimization has been a hot research direction for researchers in recent years. Some researchers have prepared composite bipolar plates that meet the DOE performance requirements. The direction of structure optimization is very diversified, and the performance of composite bipolar plates can be effectively improved by the dispersion optimization of graphite–resin, the optimization of the conductive pathway, and the optimization of the particle surface. The means of implementation are also different; layer-by-layer pressing, resin modification, filler surface functionalization, *etc.*, are all feasible methods. In conclusion, the effect of structure optimization on the performance of composite bipolar plates, especially in the actual operation of fuel cells, can continue to be studied in depth. The development of new structural optimization methods with practical potential, as well as the combination of well-designed structures with the addition of nanofillers is also a large room for improvement in the performance of bipolar plates in future studies.

## 4 Challenges, outlook and conclusions

As one of the main structural materials of PEMFC, the performance and cost of bipolar plates have an important impact on the practicalization of PEMFC. As a class of potential bipolar plate materials, composite bipolar plates have the advantages that graphite bipolar plates and metal bipolar plates do not have, and compared with the lower corrosion resistance and durability of metal plates,^123^ composite bipolar plates have better corrosion resistance and machinability, but at present, there are still problems such as difficulty in balancing the mechanical properties and electrical conductivity, high cost, and low overall performance.^124,125^

In order to solve the above problems, the addition of high-performance nanofillers and the optimization of the internal structure of the bipolar plate are mainly used to enhance its performance. Nano-fillers can effectively enhance the electrical conductivity and mechanical properties of the composite bipolar plates, but there are also the following challenges^36,66,77,99,126,127^ to be solved:

(1) Nano-fillers are difficult to disperse and easy to agglomerate. Therefore, it is important to choose a suitable method for adding and dispersing nanofillers.

(2) Nanofillers are costly, so it is necessary to rationally choose the type of nanofillers and the amount of nanofillers added to ensure that the overall cost is reasonable.^126^

(3) Poor wettability of the nanofillers with the resin, unstable interfacial bonding and low percolation threshold. Therefore, the use of dispersant, interfacial modification, and adequate dispersion can be considered to improve it.

(4) The shape of the nanofiller is also important in influencing the performance of the composite bipolar plate, and the appropriate type of nanofiller should be selected in combination with the graphite/resin void structure and other factors.

Another possible direction is to design the structure of the composite bipolar plate so that higher performance can be achieved using the same formulation without additional fillers. Currently, the main optimization directions are optimization of the distribution morphology during the molding process of graphite and resin, optimization of the internal conductive pathways, and optimization of the interface between particles. In future research, more comprehensive studies can be conducted along these lines, or new structural optimization methods with better results and lower costs can be developed. It is also important to note that structural optimization can often be used in conjunction with nanofillers as well to obtain higher performance.

In conclusion, this paper reviews the research papers related to PEMFC composite bipolar plates and analyzes and summarizes the research results of composite bipolar plates in recent years. Currently, nanofillers and structural optimization are mainly used to enhance the performance of composite bipolar plates, among which MWCNTs, CB, GNP, and CNFs are potential nanofillers, but at this stage, nanofillers still suffer from the problems of easy agglomeration, wettability, and poor dispersion, which need to be overcome in the future research. The current research on structure optimization mainly focuses on the dispersion morphology between graphite/resin, the construction of conductive pathways inside the bipolar plate, and the improvement of the properties of the contact interface of different materials. Structure optimization in a reasonable way is not only easy to operate in the process but can also further improve the performance of the composite bipolar plate under the same formula.

## Author contributions

Conceptualization, Z. X. and W. L.; methodology, W. L.; software, W. L.; formal analysis W. L.; investigation, W. L.; resources, Z. X.; writing—original draft preparation, M. L.; writing—review and editing, Z. X. and W. L.; visualization, W. L.; supervision, Z. X.; project administration, Z. X.; funding acquisition, Z. X. All authors have read and agreed to the published version of the manuscript.

## Conflicts of interest

There are no conflicts to declare.

## Supplementary Material
